# The Possible Associations between Tauopathies and Atherosclerosis, Diabetes Mellitus, Dyslipidemias, Metabolic Syndrome and Niemann–Pick Disease

**DOI:** 10.3390/diagnostics14161831

**Published:** 2024-08-22

**Authors:** Aleksandra Fryncel, Natalia Madetko-Alster, Zuzanna Krępa, Marek Kuch, Piotr Alster

**Affiliations:** 1 Students’ Scientific Circle, Department of Neurology, Mazovian Brodno Hospital, Medical University of Warsaw, Ludwika Kondratowicza 8, 03-242 Warsaw, Poland; 2 Department of Neurology, Mazovian Brodno Hospital, Medical University of Warsaw, Ludwika Kondratowicza 8, 03-242 Warsaw, Poland; natalia.madetko@wum.edu.pl (N.M.-A.); piotr.alster@wum.edu.pl (P.A.); 3 Department of Cardiology, Hypertension and Internal Disease, Mazovian Brodno Hospital, Medical University of Warsaw, Ludwika Kondratowicza 8, 03-242 Warsaw, Poland; zuzia.krepa@gmail.com (Z.K.); marek.kuch@wum.edu.pl (M.K.)

**Keywords:** tauopathies, diabetes mellitus, dyslipidemias, atherosclerosis, Niemann–Pick disease, metabolic syndrome, carotid artery stenosis, endothelial dysfunction

## Abstract

Clinical evaluation and treatment of tauopathic syndromes remain a challenge. There is a growing interest in theories concerning their possible associations with metabolic diseases. The possible connection between those diseases might be linked with cerebrovascular dysfunction. The endothelial cell damage and impairment of the blood–brain barrier observed in atherosclerosis or diabetes may play a role in contributing to tauopathic syndrome development. Additionally, the inflammation evoked by pathological metabolic changes may also be involved in this process. Multiple cases indicate the coexistence of metabolic disorders and tauopathic syndromes. These findings suggest that modifying the evolution of metabolic and cerebrovascular diseases may impact the course of neurodegenerative diseases. Obtained data could indicate the possible benefits of introducing routine carotid artery sonography, revascularization operation or antihypertensive medications among patients at high risk for tauopathies. This review has identified this understudied area, which is currently associated with several diseases for which there is no treatment. Due to the pathomechanisms linking metabolic diseases and tauopathies, further investigation of this area of research, including cohort studies, is recommended and may provide new pharmacological perspectives for treatment.

## 1. Introduction

**Figure 1:**
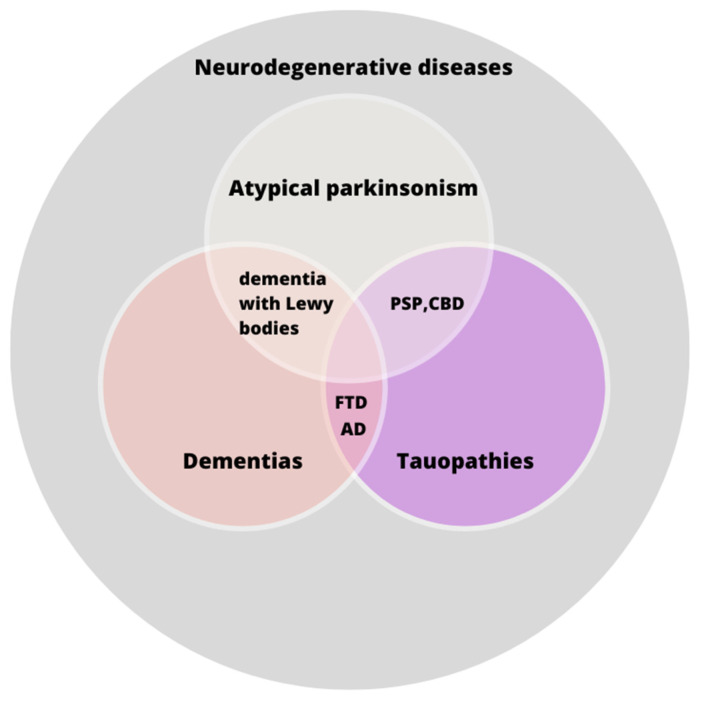
A graphical representation of the relationships between specific tauopathies and their shared classification within broader groups. AD—Alzheimer’s disease; CBD—corticobasal degeneration; FTD—frontotemporal dementia; PSP—progressive supranuclear palsy. The relationships depicted in the figure illustrate the intricate nomenclature associated with tauopathic disorders. While certain tauopathies are classified as forms of dementia, it is noteworthy that PSP and CBD do not fall within this category.

Tauopathies comprise multiple disorders, among which Alzheimer’s disease (AD), frontotemporal dementia (FTD), progressive supranuclear palsy (PSP), corticobasal degeneration (CBD) and argyrophilic grain disease can be mentioned [1]. A more detailed representation of the relationships between these diseases and their respective groups can be found in Figure 1. AD is the most prevalent form of dementia in general, accounting for 60% to 80% of all cases [2,3]; other tauopathies are relatively uncommon. This group is associated with the accumulation of tau, a protein that plays a key role in the stabilization of the microtubules in the axons and consequently in axonal transport [4]. Tauopathies are more commonly observed among advanced-aged patients [5]; however, the incidence of frontotemporal dementia (FTD) is observed at an earlier age than the other diseases from this group [6]. The most common symptoms associated with tauopathic disorders include dementia, language difficulties, psychiatric disorders and postural instability [6,7,8]. However, tauopathies are affected by overlapping clinical manifestations, e.g., PSP and CBD, which may cause difficulties in obtaining a proper diagnosis. The questionable indications of these diseases are highlighted in the recently indicated diagnostic term, “4-repeats tauopathies” [9]. The treatment is largely symptom-based, as the underlying causes of the diseases remain poorly understood, and there are currently almost no effective causal therapies.

The pathomechanism of these diseases is not sufficiently described. Furthermore, there is a growing interest in the search for tools enabling earlier diagnosis. Recently, neurodegeneration was described in the context of its possible links with nutrition [10] and hormonal imbalance [11]. Additionally, the potential influence of metabolic diseases such as diabetes, atherosclerosis or dyslipidemias on inflammatory and endothelial changes was evaluated [12,13,14]. This hypothesis is of particular interest, as many studies have already indicated the important role of neuroinflammation in neurodegeneration [15,16]. As will be demonstrated in the following sections, our findings appear to align with this theoretical framework. Dementia associated with vascular abnormalities may be also related to the pathogenesis of neurodegenerative disorders and is possibly relevant to metabolic–tauopathic associations. This hypothesis states that harmful changes in the cerebral vascular network could contribute to the development of tauopathic diseases. The presented theory has been more widely explored in AD, and it was suggested that the pathologies may coexist [17]. Recent studies showed that CBD may also be associated with vascular changes [18]. The aforementioned concepts may indicate the potential predisposition of patients with metabolic diseases to developing tauopathic disorders. If proven to be accurate, this novel theory could facilitate more effective diagnosis, treatment, and even prevention of neurodegenerative diseases.

The goal of this work is to summarize and speculate about the possible correlations between tauopathies and common metabolic disorders such as dyslipidemia, atherosclerosis, diabetes and rarer diseases, for example Niemann–Pick disease.

## 2. Methods

Two databases were used in this review: PubMed and Wiley Online Library. The analyzed types of articles included reviews, systematic reviews, meta-analyses, clinical trials, case reports and books chapters. The searched phrases included the following: “Alzheimer’s disease” AND “carotid artery stenosis”; “Frontotemporal dementia” AND “carotid artery stenosis”; “Progressive supranuclear palsy” AND “carotid artery stenosis”; “Corticobasal degeneration” AND “carotid artery stenosis”; “tauopathies” AND “carotid artery stenosis”, “Frontotemporal dementia” AND “diabetes”; “Progressive supranuclear palsy” AND “diabetes”; “Corticobasal degeneration” AND “diabetes”; “tauopathies” AND “diabetes”; “Frontotemporal dementia” AND (“dyslipidemia” OR “cholesterol”); “Progressive supranuclear palsy” AND (“dyslipidemia” OR “cholesterol”); “Corticobasal degeneration” AND (“dyslipidemia” OR “cholesterol”); “tauopathies” AND (“dyslipidemia” OR “cholesterol”); “Frontotemporal dementia” AND “Niemann Pick disease”; “Progressive supranuclear palsy” AND “Niemann Pick disease”; “Corticobasal degeneration” AND “Niemann Pick disease”; “tauopathies” AND “Niemann Pick disease”; “Alzheimer’s disease” AND “metabolic syndrome”; “Frontotemporal dementia” AND “metabolic syndrome”; “Progressive supranuclear palsy” AND “metabolic syndrome”; “Corticobasal degeneration” AND “metabolic syndrome”; “tauopathies” AND “metabolic syndrome”; “Alzheimer’s disease” AND “abdominal obesity”; “Frontotemporal dementia” AND “abdominal obesity”; “Progressive supranuclear palsy” AND “abdominal obesity”; “Corticobasal degeneration” AND “abdominal obesity” and “tauopathies” AND “abdominal obesity”. After excluding publications in languages other than Polish and English, those without the necessary relevance to the topic, those with limited access to the whole text and those which appeared from both databases in the search results, at the moment of writing (August 2024), there remained 92 articles showing associations between tauopathies and chosen factors.

## 3. State of the Art

### 3.1. Dyslipidemias and Tauopathies

Dyslipidemia is a term for many various medical conditions for which a common feature is an imbalance in the amount of lipids such as low-density cholesterol (LDL), high-density cholesterol (HDL) or triglycerides [19]. It is also linked with changes in apolipoproteins, which are the crucial components of lipoproteins that transport HDL, LDL and triglycerides in the body. Dyslipidemias are also well known to correlate with neurological conditions [20,21], which possibly aggravate them or even contribute to their development.

Many studies focused on exploring the issue of Apolipoprotein E (ApoE), which is recognized as a crucial factor in the central nervous system. It is a component of chylomicrons, HDL and VLDL (very-low-density protein), and is responsible for the transport of cholesterol from the blood to the neurons and for reducing free lipid concentrations in the blood [22]. ApoE is produced mainly in the liver and has three isoforms: E2, E3 and E4; the correlation between ApoE4 and AD has been more extensively investigated. In their work, Serrano-Pozo et al. and Koutsodendris et al. suggested that ApoE4 works as a trigger of Aβ accumulation by promoting its transformation from an Aβ peptide into fibers, inhibiting its clearance and enzymatic degradation [23,24]. Furthermore, ApoE4 was found to be involved in tau-pathology. The precise mechanisms of how this apolipoprotein causes this pathology are still debatable, but it is speculated that it involves dysregulation of tau phosphorylation, which could further lead to neuronal loss [25]. ApoE4 can also increase blood–brain barier (BBB) permeability and lead to neuronal loss. All mentioned processes could lead to the development of AD. Dilliott et al. proved a similar connection between increased E2 isoform levels and the occurrence of FTD [26]. However, contrarily, the results of a study performed on a large group of patients with PSP (*n* = 202) and CBD (*n* = 41) did not reveal a correlation between increased levels of ApoE and these tauopathies [27].

Several other studies concentrated on measuring the LDL and HDL cholesterol levels in the blood of patients with and without tauopathy. One of them, performed on patients with FTD, revealed that the average HDL cholesterol level did not significantly differ in comparison with the control group but that there was a significant difference in the case of LDL cholesterol [28]. These results are likely to confirm a positive association between the level of LDL cholesterol and the incidence of FTD. Another study delivered similar results, as in a group of patients with the behavioral variant of FTD, there was a significant difference in LDL levels and additionally in total cholesterol levels in contrast with those of the control group [29]. Additional confirmation was based on measurements of the level of LDL-C (which is an amount of cholesterol carried by LDL) in various neurodegenerative diseases, including PSP. Weng et al. discovered that among patients with PSP, the LDL-C levels were decreased, and they presented a lower LDL-C/HDL-C ratio, which was associated with worse results on tests such as the NMSS (non-motor symptom scale) and PSPRS (PSP rating scale) [30].

Other scientific groups tried to correlate the incidence of general dyslipidemia and tauopathies. In one of them, 47.6% of patients with FTD also had hypercholesterolemia; similar results were also obtained for PSP (46.9%) and CBD (46.9%) [31]. Another revealed a significant increase in triglyceride levels among patients with the behavioral variant in comparison with the control group [32]. Similar results were obtained by Phan et al., who also showed increased triglyceride levels in the serum of FTD patients compared to controls [33]. In contrast, Golimstok et al. showed that 57% of patients with FTD had dyslipidemia, and similarly, so did 54.7% of non-FTD patients [34]. The presented results showing dyslipidemia as a possible comorbidity may suggest its association with FTD. The lack of studies performed on patients with other tauopathies prevents us from proposing a specific thesis about their connections with dyslipidemia, but as they share a common pathogenetic mechanism with FTD, their coexistence with dyslipidemia is likely.

### 3.2. Atherosclerosis and Tauopathies

**Figure 2:**
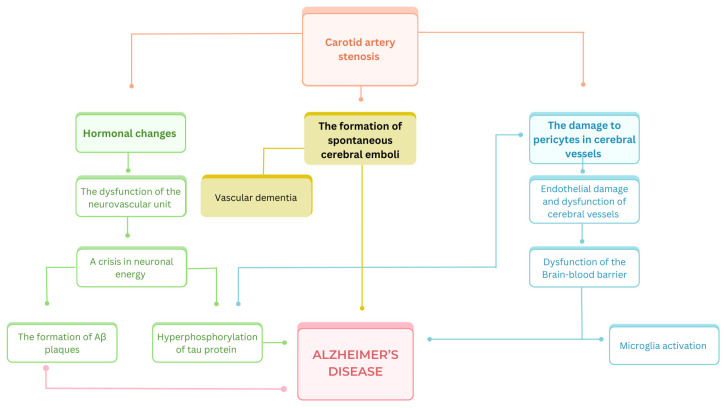
The possible associations between carotid artery stenosis and Alzheimer’s disease. Aβ—amyloid beta. As previously outlined, there are three principal theories pertaining to the mechanisms by which carotid artery stenosis may precipitate the development of Alzheimer’s disease (AD).

Atherosclerosis is a vascular disease caused by the formation of fatty plaques consisting of lipids and cholesterol in the intima–media, which leads to an abnormal thickness of vessel walls [35]. These pathogenic processes can take place in the carotid artery, resulting in stenosis, which is a well-established factor in the development of cerebral ischemic disease and ischemic stroke [36,37,38]. It is hypothesized that neurodegenerative diseases including tauopathies can be associated with carotid artery stenosis. This pathological condition is linked with endothelial damage in the small cerebral vessels, degradation of pericytes, and dysfunction of the BBB [39,40,41]. These mechanisms lead to white cerebral matter damage and superactivation of the microglia [42], and consequently may activate pathological processes, evolving into AD [43,44]. Interestingly, it is hypothesized that the formation of amyloid-β plaques can additionally contribute to endothelial damage [45] and the progression of stenosis. The formation of spontaneous cerebral emboli is also considered a factor contributing to the progression of AD [46,47]. In contrast to large emboli leading mainly to stroke or transient ischemic attack (TIA), smaller, recurrent emboli could cause progressive cerebral damage [47]. It was suggested that due to the vascular damage caused, frontostrial pathways could be damaged and therefore lead to one of the AD symptoms—depression [48]. Emboli can be the consequence of chronic carotid artery stenosis, and they were detected in more than a half of patients with >70% carotid artery stenosis within several hours of monitoring tests being conducted [47]. Li et al. proved that carotid artery stenosis also promotes an increase in levels of interleukin 18 (IL18), which is a marker of the inflammation process [49]. Additionally, a similar observation was made for patients with AD [42]. This supports the theory that the release of this cytokine, caused by the narrowing of the carotid artery, could potentially lead to neuroinflammation and contribute to AD development. Vascular changes could also be associated with AD in terms of its other pathogenetic component—neurofibrillary tangles formation. It was proven that in contrast to previously discussed hypotheses, there was no significant increase in beta-amyloid plaque formation among patients with carotid artery disease [50]. The aforementioned conceptualisations, which give rise to the AD development, are also illustrated in Figure 2.

The main issue resulting from the above-described association is the possible use of carotid artery ultrasonography as a non-invasive diagnostic procedure feasible in highlighting risk factors for tauopathies [51,52]. Therefore, revascularization of the internal carotid artery could be considered a form of prevention of the development of AD [53,54] and probably other tauopathic diseases too. Nevertheless, it is still unknown to what extent it is possible to perform this procedure on patients with asymptomatic carotid artery stenosis [55]. There is no information on how effective these surgeries are in the prevention of the development of AD. One study suggested that revascularization has no positive impact on cognitive functions [56]. In multiple papers, the effect of the mentioned procedure was too small to ascertain whether or not this operation could decrease the risk of developing AD. The current literature lacks follow-up studies verifying the rate of patients who develop AD after revascularization. Hypertension is one of the modifiable risk factors of atherosclerotic cardiovascular disease. An association between elevated blood pressure and dementia has also been shown. In one of the analyzed studies, it was been suggested that treatment with antihypertensive drugs may be beneficial among high-AD-risk patients [52]. Although these data are promising, more studies are required to confirm them.

### 3.3. Diabetes Mellitus and Tauopathies

Diabetes mellitus (DM) is defined as a group of diseases characterized by hyperglycemia, which is caused by either a decreased secretion of insulin (mainly type 1 of DM) or an impaired response of cells to this hormone (mainly type 2 of DM) [57,58]. Epidemiological evidence shows associations between diabetes and cognitive dysfunctions [59]. Most of the studies were focused on AD and proved that type 2 DM is associated with brain atrophy. Additionally, mice with induced diabetes presented an increase in Aβ and in the hyperphosphorylation of tau protein—features associated with AD development [59]. Similar, observations were made for patients with a deficiency of insulin; in this study, spatial memory impairment was also observed [60,61]. As the number of papers that concentrated on the link between DM and AD was high, this article is focused on rarer tauopathies with the exception of AD.

Various researchers presented hypotheses on the possibly higher risk of the development of tauopathies such as FTD or CBD among patients with DM [34,62]. The first of them claimed that vessel structure changes, caused by DM, also apply to to small cerebral vessels that form the BBB [63,64]. Loss of the protective role of BBB can consequently contribute to the development of neurodegenerative disorders. The mechanisms of this phenomenon are better described in AD; however some of them share a common pattern with other tauopathies. The consequences of a disrupted BBB, such as cerebral microbleeds, may change the response to systemic inflammation, and impaired glucose transport could contribute to the development of tauopathic diseases [65,66]. Other authors also point out the fact that DM may also cause inflammation in the cerebrum [12,67], which could lead to neuronal death and the development of neurodegenerative changes, as noticed in tauopathies [68].

The link between DM and tauopathies is due to various mechanisms. Firstly, the expression of the enzyme GSK3β in mice, which is a proline-directed serine-threonine kinase that was initially described as an enzyme responsible for phosphorylation and therefore the inactivation of glycogen synthase, was studied [69]. Further studies proved that GSK3β is hyperactive in patients with AD and is involved in tau phosphorylation and neuronal degenerative changes [70,71]. The fact that diabetes and dementia have this feature in common could indicate the possible correlation between them. There is also experimental evidence that neuronal insulin signaling, dysregulated in DM, also impairs cognitive functions, and is associated with tauopathies [72]. Confirmation of the “inflammatory theory” comes from another experimental study [73], which proved that NOD-like receptor pylorine NLRP3 has a crucial role in both diabetes and neurodegenerative changes, which could also reflect the possible tendency to develop neurodegenerative disorders in patients with DM. Another study explored the possible connection between BBB damage caused by diabetes and the presence of FTD [64]. The authors used serum albumin quotient (Qalb), which represents the ratio of cerebrospinal fluid (CSF)/plasma albumin and is treated as a marker of BBB damage. In this work, the previously discussed theories supported that chronic inflammation and oxidative stress caused by DM can lead to endothelial and vascular changes, including changes in the BBB [74]. As evidence of this hypothesis, the authors referred to their previous works using the body max index (BMI), where a value over 25.0 indicates that the patient is overweight. In these papers, they observed that its increase in middle age is predictive of a higher Qalb ratio. Furthermore, high BMI is proven to be one of the predictors of type 2 diabetes [75]. It was also verified that this value was increased in FTD in coexistence with DM. In this study, markers of endothelial dysfunction such as the ratios of VEGF/sVEGF, ICAM-1 and VCAM-1 were increased in patients with FTD and diabetes in comparison with those of patients without DM. This observation could be explained by peripheral vascularity dysfunctions, caused by diabetes. In summary, this study also suggested the possible role of diabetes in damaging the BBB and initiating degenerative changes.

Apart from experimental studies, some authors tried to estimate the number of patients with tauopathies and diabetes as a comorbidity to establish and eventually prove the possible connection between them. The results of these studies provide contrary outcomes and can be divided into two types: those contraindicating and supporting the hypothesis of a higher prevalence of DM amongst patients with tauopathic disorders.

One of the discussed tauopathies in this context was PSP. Research based on an examination of 892 patients revealed that only 24.9% had DM [76]. In contrast is another study that engaged with PSP patients, which despite having statistical insignificance, noted a positive tendency between PSP and DM [77]. The outcome of this study agree with the conclusions obtained from a German multicenter observational study showing that amongst 335 PSP patients, 13.4% also had DM (in contrast with 4.7% of the 275 control patients) [78]. The next study with similar results was based on electronic medical records from general practices; 152 PSP patients and 3.122 matched controls were included [79]. The results showed that amongst the PSP patients, 8.3% had DM type 1 only, 87.5% had DM type 2 only and 4.2% had both types of DM.

In the case of FTD, one study showed that out of 63 patients with this tauopathy, only 31.7% had diabetes; however, this study confirmed that DM occurs more frequently in patients with FTD than in patients with non-FTD [80]. The confirmation of a possible correlation could be exemplified by a study performed by the authors of [34]. In this research, 100 patients with FTD were compared with 200 control individuals to identify comorbidities, including DM. The results showed that the prevalence of DM was significantly higher among FTD patients than in the control population (39% vs. 22.6%).

Interestingly, results of studies on multiple tauopathies such as the one performed by [31] did not support the hypothesis about a possible association between them and diabetes. In this research, based on information from the National Alzheimer’s Coordinating Center (NACC, USA), data ere gathered from a wide group of patients with FTD (778 patients), PSP (63 patients) and CBD (80 patients), including diabetes as a comorbidity. It was shown that DM occurred in only 9.5% of FTD patients, 10.9% of PSP patients and 4.9% of CBD patients.

As presented, there is a lack of consensus about the possibly higher presence of DM among patients with tauopathies. The probable explanation of this association could be that patients suffering from DM had decreased brain glucose levels, which may have led to a decrease in oxidative phosphorylation and, consequently, an increase in reactive oxygen species and then tau pathology [79]. However, another theory states that DM prevalence increases with age, and a similar tendency was noticed among most of the tauopathies. Consequently, studies comparing patients with tauopathies with an similar, elderly control group have failed to reveal statistically significant differences. This leads to the suggestion that the coexistence of DM and tauopathy is a matter of chance Resulting from older age, as has been commonly observed. 

### 3.4. Metabolic Syndrome and Tauopathies

The global prevalence of metabolic syndrome (MetS) is estimated in one of study to be 63.3% in 2023, making it a significant public health concern [81]. MetS is used to describe a situation in which a patient presents with at least three of the following conditions: abdominal obesity, hypertension, insulin resistance, high blood triglycerides and low HDL levels [82]. The impact of metabolic syndrome on the development of cardiovascular disease is well established. However, recent research has also investigated its potential influence on tauopathic diseases. Given that MetS is a combination of pathological conditions, including diabetes, dyslipidemia and atherosclerosis, it is probable that it also shares their pathological mechanisms in terms of the development of tauopathies. Consequently, this aspect was omitted, and below, only visceral obesity is discussed in this context.

It has been demonstrated that abdominal obesity is associated with low-grade inflammation, which can be attributed to the elevated expression of pro-inflammatory cytokines due to an excess of adipose tissue [83]. Furthermore, the activation of distinct inflammatory pathways in obesity [84] has been demonstrated to exert a deleterious influence on cognitive functions, thereby increasing the risk of tauopathy development. Additionally, obesity has been linked to the onset of hypoadiponectinemia and leptin resistance, which may subsequently intensify the accumulation of Aβ and tau phosphorylation [85]. It can be reasonably deduced that the aforementioned mechanisms may ultimately result in the progression of neurodegenerative disorders.

The theoretical assumptions regarding the potential associations between MetS and tauopathies have been subjected to epidemiological studies, particularly in the context of Alzheimer’s disease. The results of these studies are inconclusive. A 2021 meta-analysis revealed a significant correlation between metabolic syndrome and AD. It was unexpected that, in contrast to other components of metabolic syndrome, increased waist circumference was identified as a protective factor against the development of AD [86]. This revelation was explained as the effect of reverse causality. Another study from 2023 based on data obtained from UK Biobank revealed that MetS was associated with a 12% increased risk of dementia [87]. In contrast to the mentioned research, results from an older metastatic study from 2019 demonstrated no statistically significant correlation between MetS and the incidence of AD and dementia. However, the study did indicate that metabolic syndrome was associated with an increased risk of pure vascular dementia [88]. Also, 2 years later, Rodriguez-Santiago et al. found no statistical association between MetS and dementia in their study [89]. A review of the literature revealed no studies that specifically addressed the epidemiological correlation between MetS and other tauopathies, such as FTD or CBD. In light of the cited studies, it is challenging to draw a definitive conclusion regarding this epidemiological correlation, which underscores the urgent need for further research in this area.

In addition to epidemiological studies, attempts have been made to identify a genetic correlation between MetS and tauopathies. However, the findings of He et al. in 2023 did not indicate any associations of MetS and its components with an increased risk of developing AD, FTD or other forms of dementia [90].

The aspect is most closely associated with metabolic syndrome is dietary intake. It has been demonstrated that the diets typical in Western countries, which are regarded as unhealthy, can result in the development of metabolic syndrome. This may occur because of the diet inducing obesity, hypercholesterolemia or hyperglycemia, or causing adverse changes in the composition of the gut microbiota. Subsequently, these conditions may result in metabolic and systemic inflammation, which could subsequently contribute to the progression of tauopathies such as AD [91,92]. This correlation is particularly noteworthy and underscores the necessity to reframe our understanding of nutrition as a critical determinant in the pathogenesis of neurodegenerative disorders.

### 3.5. Niemann–Pick Disease and Tauopathies

In addition to the common disorders described above, some rarer metabolic diseases have been suggested to be associated with tauopathies. One of these is Niemann–Pick disease (NPD), a rare inherited lysosomal storage disease characterized by an inability to metabolize lipids within cells, leading to cell dysfunction and death [93]. This disorder affects many organs in the body, causing splenomegaly, hepatomegaly or most importantly in this paper, changes in the cerebral cortex and subcortical structures, leading to neuronal damage, to name a few outcomes [94]. There are three types of NPD, A, B and C, with C being the most common. Genes with mutations that are involved in the development of the disease are SMPD1 for types A and B, and NPC1 and NPC2 for type C [95]. Over the last decade, it has been hypothesized that dysregulation of the NPC1 and NPC2 genes may follow a common pattern and coexist with several cerebral and brainstem diseases. These genes encode transport proteins involved in the transport of lipids across membranes, which in the case of NPC1 or/and NPC2 mutation, leads to dysregulation of lipid balance [96].

Boenzi et al. aimed to investigate the possible presence of NPC1 or NPC2 variants in patients with PSP, but no changes were found [97]. Other authors have also rejected the idea of a possible association between NPD and tauopathies. The first study included 133 patients with FTD, 94 with PSP, 563 with Parkinson’s disease and 846 healthy controls. Variants of the NPC1 or NPC2 gene were found only in Parkinson’s disease patients, but not in a statistically significant number of them (1.1% vs. 0.8% in the control group). There were no similar observations in the two other groups studied [98]. However, there are several case reports describing patients with both NPD and tauopathies. The authors reported a patient with NPD clinically manifested as CBD [99] and patients with NPD and PSP [100,101,102]. Voinea et al. also presented a case of a patient with NPD and FTD [103]. Furthermore, a case report from 2023, which details the case of a 65-year-old male with NPD and severe aortic stenosis, is worthy of note. Despite the absence of any tauopathies, this example serves to illustrate the potential comorbidities of metabolic disease and the efficacy of transcatheter aortic valve implantation (TAVI) as a treatment for aortic stenosis [104].

## 4. Clinical Implications 

### 4.1. Discussion—Common Mechanisms of Metabolic Diseases in Tauopathy Pathogenesis

Table 1 presents a short summary of the previously described potential associations between metabolic disorders and tauopathies. In all discussed diseases, dyslipidemias, arteriosclerosis, DM, MetS and NPD, there was a common pattern noticed by authors that potentially contributed to the development of neurodegenerative disorder, and therefore, potentially, atypical parkinsonian disorders too. One of the frequently mentioned theories was the inflammation theory. In atherosclerosis, the formation of plaques contributes to damage to the endothelium and provokes inflammatory conditions, which are evidenced by increased inflammatory markers such as Il18. The precise triggering mechanism of the inflammation in DM is poorly understood; however, proposed theories include oxydative stress or amyloid deposition in the pancreas [105]. Dyslipidemia and NPD share the same possible pathway for the development of tauopathies because the imbalance in lipid metabolism can also be an inflammatory factor. Consequently, an induced inflammatory condition can lead to neuronal dysfunction, the production of abnormal proteins and the development of tauopathies. It is crucial to acknowledge that a significant number of the studies upon which we based our hypothesis were not specifically designed to investigate the association between tauopathies and metabolic diseases. Nevertheless, the coexistence of these disorders was observed in many clinical cases.

### 4.2. The Most Recent Data from 2024

The recent progress concerning the issue presented in this manuscript was additionally analyzed in the context of advances from the last 12 months. The methodology of the search was identical to that described in the section entitled “Methods”, with the exception of the filter used for the publication date, which was set to “1 year” in PubMed (Medline) and “last 12 months” in Wiley Online Library.

Initially, some authors sought to further examine the potential links between tauopathies and metabolic disorders. One illustrative example is the work of Soleymani et al., who presented a review with the objective of exploring the associations between glycosylated hemoglobin (HbA1c) and brain structure and function [106]. HbA1c is a marker that is commonly used for the assessment of glucose intolerance. High levels of HbA1c serve as an indirect indicator of poor treatment of diabetes [107]. It has been demonstrated that HbA1c levels might not only influence the volume of white matter and the hippocampus, but also affect a range of cerebral functions [108]. Although the evidence is inconclusive, the authors hypothesize that fluctuations in blood sugar levels, which result in changes in HbA1c levels, may contribute to hippocampal atrophy and, consequently, the development of AD or other forms of dementia. Another study has proposed a potential link between AD and diabetes owing to vacuolar adenosine triphosphatases (v-ATPases), which regulate lysosomal acidification and glycolipid metabolism. It was demonstrated that these enzymes may facilitate glycolysis and insulin secretion, while also reducing the deposition of Aβ. It can therefore be surmised that impairments in v-ATPase function may result in the development of both DM and AD [109]. Once again, the role of insulin resistance in evoking AD was reported and pointed out as an essential pathomechanism shared between this disease and DM [110,111]. An intriguing hypothesis regarding the link between MetS and Alzheimer’s disease was put forth by Kim et al., who conducted experimental studies demonstrating that elevated blood glucose levels observed in MetS patients not only induced insulin resistance but also promoted amyloid precursor protein phosphorylation via extracellular vesicles. These events may ultimately lead to tau phosphorylation and the subsequent development of tauopathies [112]. A further study demonstrated that, in individuals with metabolic syndrome, the accumulation of apoE4 can result in a reduction in white matter volume and an increased risk of neurodegeneration [113]. This concept provides deeper insights into the impact of lipid dysregulation on the potential for tauopathies to develop. In a separate study conducted in China, it was demonstrated that individuals with MetS and its associated components exhibited elevated levels of Aβ42, which is also commonly used as an AD marker, in their serum [114]. The same phenomenon was also observed by other authors [112]. This finding may therefore serve to confirm the potential association between MetS and the development of AD. Furthermore, elevated levels of secreted phosphoprotein I (SPPI), insulin-like growth factor 1 (IGF1), vascular endothelial growth factor receptor 1 (FLT1) and CD44 in both MetS and AD may also be treated as potential markers of the two conditions [115].

The second group of studies aimed to statistically demonstrate a higher incidence of tauopathy in patients with specific metabolic disorders. One of the studies sought to examine the potential correlations between lifestyle-related illnesses and FTD and AD. It is noteworthy that patients with FTD were less likely to have a history of DM than patients with AD. Concurrently, no statistically significant differences were observed in the prevalence of hypertension and dyslipidemia [116]. As AD is the most prevalent form of dementia, these findings indirectly indicate the detrimental impact of DM on the progression of it. A study published in 2024 demonstrated significant correlations between ApoE, hypertension and dementia, including FTD and AD, separately. However, the associations between diabetes and dementia were found to be inconsistent [117]. Another author concentrated their research on PSP and its comorbidities. It was demonstrated that the prevalence of DM and diseases of the circulatory system was higher among PSP patients than in the control group. It is noteworthy that metabolic diseases were not identified as more prevalent in the experimental group. Additionally, distinctions were observed between the various forms of PSP, namely progressive supranuclear palsy variants (vPSP) and progressive supranuclear palsy–Richardson syndrome (PSP-RS). Hypertensive and metabolic diseases, as well as diabetes, were observed to be more prevalent in PSP-RS than in vPSP [78]. In the case of components of metabolic syndrome, the latest meta-analysis from 2024 reached the significant conclusion of a differentiation between metabolically healthy obesity and metabolically unhealthy obesity. The findings indicated that patients in the first group exhibited a reduced probability of developing AD and other forms of dementia. Conversely, no notable association with dementia onset was observed in the second group. It is noteworthy that the risk of AD was elevated in non-obese individuals with metabolic dysfunction [118]. Furthermore, a new case report was published in which a 46-year-old male with NPD was observed to develop PSP [119].

This new information from the past 12 months provides partial confirmation of the previously discussed hypothesis regarding the associations between metabolic diseases and tauopathies. Some of the findings also provided new insights into the potential nature of these associations. In general, it appears that the metabolic component is of great importance for a more profound comprehension of the pathogenesis of tauopathies and the prospective forms of their prevention.

### 4.3. The Present and Future Medications for Metabolic Diseases and Their Impact on Tauopathies

One of the key reasons for investigating the associations between tauopathies and metabolic diseases is to develop new strategies for preventing neurodegeneration by treating underlying diseases such as diabetes or dyslipidemia. The concept of using pharmacological approaches linking the treatment of metabolic disorders with the prevention of tauopathic diseases is a relatively novel one, and the paucity of existing studies precludes any definitive conclusions regarding their efficacy. Nevertheless, the current medications employed in the treatment of these diseases may also prove effective in slowing the progression of tauopathies. In this context, particular attention is warranted regarding the potential role of sodium-glucose cotransporter-2 (SGLT2) inhibitors [120], thiazolidinediones [121] and dipeptidyl peptidase 4 (DPP-4) inhibitors [122] which are used in the management of diabetes and have been demonstrated to reduce insulin resistance. This pathology is frequently observed in tauopathies and has been identified as a contributing factor to their development [72]. Consequently, these drugs may have the potential to slow the progression of tauopathic diseases. Some studies have also indicated the role of high LDL levels in tauopathies’ progression [28,29,30]. Therefore, medications designed to reduce their levels, such as statins [123] or proprotein convertase subtilisin/kexin type 9 (PCSK-9) inhibitors [124], may be beneficial not only in dyslipidemia treatment but also in the prevention of tauopathies. A new concept has emerged that aims to inhibit the action of ApoE4, a lipoprotein that plays a role in the development of tauopathies [23,24]. In 2016, the Food and Drug Administration (FDA) approved the use of antisense oligonucleotides (ASOs), a novel class of drugs that can effectively reduce the expression of the ApoE4 gene. Another class of medications with this effect comprises specific small interfering RNAs (siRNAs), which also permit the direct silencing of ApoE4 expression in the brain. Additionally, antibodies (anti-ApoE4) may also be capable of downregulating brain ApoE4 levels [125]. The targeting of this lipoprotein is purported to be a promising avenue of future therapeutic intervention. However, currently, there is a paucity of data demonstrating the long-term efficacy of these pharmacological agents.

### 4.4. Limitations

The presented theories and findings referred mainly to AD, and only a few were focused on FTD, PSP, CBD or other tauopathies. This could be a consequence of the low prevalence of these disorders, of well as of various manifestations of them, which does not allow researchers to obtain enough patients for any studies. A great example is CBD, which is claimed to be a heterogeneous disease with different symptoms [126]. Experimental verification in humans of the mentioned pathogenesis theories such as induced endothelial damage was only performed post mortem. Another significant limitation is the inability to prove the correctness of a proposed diagnosis (AD, PSP, FTD or CBD) in vivo, as definite diagnoses are based on neuropathological evaluation. Furthermore, there is a lack of prospective, cohort studies investigating the described associations, such as the coexistence of DM and tauopathy; a high number of the presented results are retrospective. This may partly affect the significance of the obtained results. These limitations indicate the strong need to develop scientific methods to make research regarding these topics possible during patient’s life too.

### 4.5. Conclusions and Future Directions

The presented work aimed to summarize and make conclusions from information about the possible associations between tauopathies and selected metabolic diseases. Based on the obtained data, it was possible to speculate that atherosclerosis manifesting as carotid artery stenosis could contribute to the development or progression of AD, with a possibility to relate this hypothesis to another tauopathy such as FTD, PSP or CBD, considering their common pathogenesis. There is also experimental and scientifically explained evidence that DM and dyslipidemias could lead to the development of tauopathies. In contrast, there is almost no confirmation about the possible association between NPD and the mentioned tauopathies, and results regarding MetS are contraindicative. Our findings suggest that the potential common mechanism linking metabolic disease with the development of tauopathic disorders may have an inflammatory background. The inflammation induced by metabolic changes may lead to neuroinflammation and neuronal changes, ultimately resulting in neurodegeneration.

Nevertheless, there are still insufficient data concerning pathomechanisms linking metabolic diseases and tauopathic disorders, which suggests the necessity for further exploration of this issue. Perhaps, conducting large cohort studies could help make clearer conclusions on this topic and open up new pharmacological perspectives for treatment. The described possible associations could have applications in clinical praxis. Firstly, the introduction of routine sonographic carotid artery sonography could allow researchers to estimate patients’ risk of developing tauopathy. Secondly, a revascularization operation might be a new form of protection from not only possible cerebral ischemia and its direct consequences but also from tauopathic disorders. Lastly, the introduction of antihypertensive, antidiabetic or regulatory lipid balance medications may be beneficial for treating not only primary disease but also for prevention from developing tauopathies. These issues are worth analyzing and may eventually contribute to better care, not only for patients already diagnosed with tauopathies, but also for those in high-risk groups.

## Figures and Tables

**Table 1:** The summary of the discussed in the article possible associations between metabolic disorders and tauopathies.

Discussed Metabolic Disorder	Associations with Tauopathies
Dyslipidemia	Imbalance in low-density lipoproteins (LDL), high-density lipoproteins (HDL) and triglycerides levels is linked to neurological conditions. The E4 isoform of Apolipoprotein E (ApoE), in particular, is associated with Alzheimer’s disease (AD) and other tauopathies. ApoE4 may lead to Amyloid beta (Aβ) accumulation and tau pathology [23,25]. The ApoE2 isoform may be associated with frontotemporal dementia (FTD) [26]; there is no strong correlation of ApoE with progressive supranuclear palsy (PSP) or corticobasal degeneration (CBD) [27]. Studies show a positive association between high LDL levels and FTD [29]; the lower LDL-C/HDL-C ratio may be linked to a worsening of symptoms in PSP [30]. A significant number of patients with FTD, PSP or CBD also have hypercholesterolemia or elevated triglycerides, suggesting a possible link [32,33].
Atherosclerosis	This is linked to carotid artery stenosis [39,40], potentially contributing to tauopathies and Alzheimer’s disease (AD) [43,44]. Carotid artery stenosis causes endothelial damage and blood–brain barrier (BBB) dysfunction, and may lead to AD through small emboli [46,47]. Carotid stenosis increases interleukin 18 (IL18) levels, suggesting a link between inflammation and AD [42,49]. There is no significant increase in Aβ plaques in carotid artery disease [50]. Carotid ultrasonography is proposed for the early detection of tauopathies [51,52]; revascularization’s effectiveness in AD prevention is unclear [53,54]. Antihypertensive treatment may reduce AD risk [52], but more research is needed.
Diabetes mellitus (DM)	Induced diabetes in mice shows increased Aβ and tau hyperphosphorylation [59]. DM may increase the risk of tauopathies like FTD or CBD [34,62] due to vessel changes and BBB disruption [63,64]. Inflammation and glucose transport impairment from DM could contribute to neurodegenerative changes [12,67,68]. The GSK3β enzyme, involved in tau phosphorylation, is hyperactive in AD and DM [70,71]. Disrupted insulin signaling [72] and inflammation [73] are common in both DM and tauopathies. PSP studies show mixed results regarding DM prevalence; however, FTD studies indicate a higher DM prevalence compared to those on non-FTD [34,80].
Niemann-Pick disease (NPD)	This disease has a hypothesized association with tauopathies due to lipid transport dysregulation caused by NPC1/NPC2 mutations [96]. A large study found no significant association between NPC1/2 mutations and tauopathies like FTD or PSP [98]; however, case reports showed a co-occurrence of NPD with CBD, PSP and FTD in individual patients [99,100,101,102,103].

## Data Availability

No new data were created or analyzed in this study. Data sharing is not applicable to this article.

## Abbreviations

AD	Alzheimer’s disease
BBB	Blood–brain barrier
BCAS	Bilateral carotid artery stenosis
CBD	Corticobasal degeneration
DM	Diabetes mellitus
FTD	Frontotemporal dementia
HDL	High-density lipoprotein
LDL	Low-density lipoprotein
MetS	Metabolic syndrome
NPD	Niemann–Pick disease
PSP	Progressive supranuclear palsy
